# Ethical challenges of seclusion in psychiatric inpatient wards: a qualitative study of the experiences of Norwegian mental health professionals

**DOI:** 10.1186/s12913-019-4727-4

**Published:** 2019-11-21

**Authors:** Espen W. Haugom, Torleif Ruud, Torfinn Hynnekleiv

**Affiliations:** 1 Innlandet Hospital Trust, Department of Acute Psychiatry and Psychosis Treatment Sanderud, 2312 Ottestad, Norway; 2 Mental Health Services, Akershus University Hospital, Box 1000, 1478 Lørenskog, Norway; 3 Institute of Clinical Medicine, University of Oslo, Box 1171 Blindern, 0318 Oslo, Norway; 4 Innlandet Hospital Trust, Department of Acute Psychiatry and Psychosis Treatment Reinsvoll, 2840 Reinsvoll, Norway

**Keywords:** Seclusion, Coercion, Ethics, Health professionals/mental health staff, Mental health services, Open-area seclusion, Shielding

## Abstract

**Background:**

Seclusion is an invasive clinical intervention used in inpatient psychiatric wards as a continuation of milieu therapy with vast behavioural implications that raise many ethical challenges. Seclusion is in Norway defined as an intervention used to contain the patient, accompanied by staff, in a single room, a separate unit, or an area inside the ward. Isolation is defined as the short-term confinement of a patient behind a locked or closed door with no staff present. Few studies examine how staff experiences the ethical challenges they encounter during seclusion. By making these challenges explicit and reflecting upon them, we may be able to provide better care to patients. The aim of this study is to examine how clinical staff in psychiatric inpatient wards describes and assess the ethical challenges of seclusion.

**Methods:**

This study was based on 149 detailed written descriptions of episodes of seclusion from 57 psychiatric wards. A descriptive and exploratory approach was used. Data were analysed using qualitative content analysis.

**Results:**

The main finding is that the relationship between treatment and control during seclusion presents several ethical challenges. This is reflected in the balance between the staff’s sincere desire to provide good treatment and the patients’ behaviour that makes control necessary. Particularly, the findings show how taking control of the patient can be ethically challenging and burdensome and that working under such conditions may result in psychosocial strain on the staff. The findings are discussed according to four core ethical principles: autonomy, beneficence, non-maleficence, and justice.

**Conclusion:**

Ethical challenges seem to be at the core of the seclusion practice. Systematic ethical reflections are one way to process the ethical challenges that staff encounters. More knowledge is needed concerning the ethical dimensions of seclusion and alternatives to seclusion, including what ethical consequences the psychosocial stress of working with seclusion have for staff.

## Background

The seclusion method is closely associated with the development of psychiatric institutions, especially the establishment of emergency units and milieu therapy. The clinical concept of seclusion implies retention of an inpatient in a bare room to contain a situation that may result in an emergency [1]. An agitated patient where there is a high risk of injury to staff or other patients may contitute such an emergency. In Norway, seclusion is legally defined as an intervention used to contain the patient, accompanied by staff, in a single room, a separate unit, or an area inside the ward [2, 3]. Concepts and methods employed in the seclusion practice in Norway include ‘open-area seclusion’, ‘segregation nursing’, ‘segregation area’, ‘quiet rooms’, ‘sheltered areas’, and psychiatric intensive care units (PICUs) [4].

The Norwegian version of seclusion is an intervention in inpatient psychiatric wards as a continuation of milieu therapy [2]. The inpatient psychiatric wards at hospitals in Norway are part of a health trust which provide access to specialized health services (secondary care). Seclusion is understood as a stimulus-limiting and protective approach towards the patient. This understanding is based primarily on psychodynamic theory and Gunderson’s five principles of milieu therapy, including the containment principle [5]. The treatment was integrated as part of milieu therapy with the presence of staff [2, 3].

Isolation in Norway is defined as short-term confinement behind a locked or closed door with no staff present [6]. The seclusion practice was meant to be an alternative to isolation as there is no locked door between the patient and staff. Under the Norwegian Mental Health Act, there is a requirement for a specific, legally based decision made by the patient’s consulting psychiatrist or clinical psychologist, and section 4.3 of the act states that seclusion can only be used when the patient has a mental condition or exhibits disruptive behaviour that makes it necessary for therapeutic reasons or consideration of other patients [6]. Therapeutic reasons may be present if it is necessary to limit the patient’s sensory impressions. Consideration for other patients is often necessary when a patient’s behaviour is very disturbing, annoying, or unfortunate for other patients, e.g., a patient that are aggressive and/or violent may be regarded as annoying for other patients [7].

In 2017 seclusion was carried out with 2517 adult patients in Norway, a rate of 58 per 100,000 inhabitants [8]. In the same year, the rate of psychiatric admissions was 640 per 100,000 inhabitants [9]. The Norwegian seclusion practice seems to have changed in the last decades and appears now to be a part of custodial care as it involves both control and isolation to different degrees [10, 11]. This has happened despite the fact that user involvement and voluntariness have been part of the main political guidelines for mental health work in the recent decades [12]. At the same time there has been an increased focus on avoiding abusive practices with a complaint and control body through The control commission. This commission is tasked with an overall responsibility for ensuring the individual’s legal certainty in dealing with the mental health services. In addition, The Parliamentary Ombudsman works to prevent torture and inhuman treatment in institutions, according to a mandate set out by the UN optional protocol to the Convention against Torture (OPCAT).

A high priority in health services worldwide is a reduction of coercive methods, including seclusion, based on an increasing emphasis on human rights, empowerment, and shared decision making [13–15]. Seclusion is an invasive clinical intervention with vast behavioural implications; it raises many ethical challenges (e.g. violation of human rights and risk of doing harm) [3, 16–19], and there are alternative strategies to minimize or replace its use. E.g. staff and patient education programs, environmental alterations, clinical supervision, sensory modulation tools and approaches, leadership and increased staff to patient ratios [18, 20–23]. Treating psychiatric patients in the least restrictive environment possible is a common aim [24]. Clinically, various forms of seclusion seem to be used as treatment options for different forms of agitation, aggressive behaviour, and disorientation [4, 25–27], and several studies have shown considerable differences in the use of seclusion among various wards and geographical areas [28–31]. This indicates a potential for quality improvement [31], and it appears that there is a major discrepancy between the widespread use of seclusion and its knowledge basis. There is a need for more knowledge as to what assessments clinical staff must carry out when making decisions regarding seclusion.

Patients often perceive seclusion negatively as it reduces their freedom. Although some patients have positive responses, more often the reactions are rage and feelings of being punished, stripped of their humanity, and overpowered [32–35]. The staff may feel it necessary and natural to carry out seclusion interventions even if there seems to be little specific research supporting a positive effect of the practice [1, 2, 36–38]. It might also be a potentially traumatizing intervention for the patients [1, 39]. Few studies examine how staff members describe, assess, and perceive ethical challenges that they encounter during coercion, including seclusion [40, 41]. By making these challenges explicit and reflecting on them, we may be able to provide better care to patients who are subject to seclusion and develop tools that better equip us to effectively use this intervention.

## Methods

### Aim and research questions

The aim of this study is to examine how clinical staff members in psychiatric inpatient wards describe and assess the ethical challenges of seclusion. The research questions are as follows:

1. What ethical challenges do clinical staff experience in practicing seclusion?

2. How does clinical staff work with these ethical challenges?

### Context

The present study is part of a project with the primary aim of creating a knowledge-based and reliable seclusion measurement tool for clinical use and research at The Norwegian Acute Psychiatry Network, Department of Research in Mental Health, at Akershus University Hospital.

### Design

This ethics sub-study is based on detailed descriptions of episodes of seclusion in psychiatric wards. It has a descriptive and exploratory design using an inductive approach [42, 43]. Using a qualitative methodology, we obtained empirical, in-depth knowledge of seclusion cases.

### Data collection, participants, and recruitment

A semi-structured form was designed for providing a written description of various aspects of seclusion in a psychiatric ward. The cases were written by the clinical staff, both therapists (psychiatrists and psychologists) and ward staff (psychiatric nurses, social educators, and social workers) who had been personally involved in the seclusion. Ward staff who participated in writing the descriptions spent several hours with the patients each day. It was emphasized that the therapists who participated should have good knowledge of the patient through regular conversations and cooperation with the ward staff. Each case was described in detail, including the patients and staff’s actions and reactions, clinical assessments, and descriptions of the physical environment and other factors that were perceived as relevant. In separate sections of the form, clinical staff was asked to describe background and rationale, objectives, seclusion measures used, ethical aspects, and termination of the seclusion. They were also asked to identify and describe how ethical considerations have influenced thoughts and actions of staff, how these have been perceived by patients and staff, and whether staff experienced value dilemmas between contradictory options.

The form was sent to 64 psychiatric inpatient wards throughout the country that had expressed interest in taking part in the project. They were all part of The Norwegian Acute Psychiatry Network and were invited because they used seclusion in their practice. Descriptions of 149 different seclusion cases were received from 57 different wards in both rural and urban areas. The vast majority of the participating wards were acute wards (about 35) and psychosis/rehabilitation wards (about 15), but some subacute and a few acute adolescent wards and psychiatric wards for the elderly were also included. Each seclusion description could be from 0.5 to 7.5 pages long, and the total material on ethical aspects consisted of approximately 25,000 words.

The patients who are described in the cases were either secluded in a regular patient room or in a secluded area. Seclusion in a regular patient room means that the seclusion took place in the same room as the patient stayed in during the rest of the admission. A seclusion area is a separate unit, section, or area where the seclusion takes place. The infrastructure varies, but there is usually more space so that the patient can be together with staff, e.g., having a living room in addition to the sleeping room. On several occasions, a small fenced area outside is described where the patient can spend time with staff in the fresh air. In total, 37 patients were secluded in regular patient rooms and 112 in seclusion areas.

The cases varied in time from 1 h to 168 days with a mean of 17 days, median of 10 days, with the time not given in 10 of the cases. In 12 cases the patients were secluded less than 24 h. There were 41 patients with a seclusion duration between 1 and 7 days and 40 patients with a duration between 8 and 14 days. In 46 cases the patients were secluded for more than two weeks.

In almost every case, the reasons given for the seclusion were therapeutic and consideration for other patients, but they also gave more specific reasons. That the patient behaviour was uncritical was used as a reason in 52 cases; E.g. the patient undresses or tells private stories (such as intimate announcements) to other patients in the common area. Exhibiting chaotic behaviour was used in 56 cases. In 19 cases the indication for seclusion was that the patient showed significantly increased activity; E.g. the patient is restless and more active than usually, contact-seeking and have a lot of questions and has difficulties sitting down. Being threatening or violent to staff was used as a reason in 60 cases, and being threatening or violent to other patients was used in 19 cases. That the patient had a high risk of suicide or serious self-injury was used in 9 cases. Note that in 56 cases, more than one reason was given for seclusion.

The sections on ethical aspects from all 149 cases were included in the study. All sections were read before the analysis to make sure that the other sections did not contain data on ethical challenges and to make sure that data in the section on ethical aspects actually was pertinent for our analysis.

### Data analysis

The method of analysis was based on Graneheim and Lundman’s [44] qualitative content analysis. The method focuses on the subject and the context. We chose an epistemological standpoint close to the text, involving specific phenomenological descriptions [45]. All descriptions were read several times to familiarize ourselves with the material and obtain a sense of whole. The purpose of this is to gain a general understanding of what the text says [46]. Meaning units were identified, condensed, and given a code. The meaning unit was identified by reading the text while searching for words, sentences or paragraphs relating to each other through their content and context. This process involved dividing the text into smaller parts. Condensing involved shortening of the unit while still preserving the core. Giving each meaning unit a code is supposed to make one think about it in new and different ways [44]. We discussed and compared the codes for differences and similarities before they were sorted into categories and subcategories, describing the content on a manifest level. This process involved abstraction, where codes, categories, and theme were created at different abstraction levels.

Ensuring that the results were sustainable and supported by empirical data enhanced credibility. This process involved comparing the subcategories, categories, and theme with the descriptions to ensure that they covered the seclusion cases as they were described by the participants [44]. The final step involved a presentation of the main theme, categories, and subcategories, exemplified by statements from the participants to show the content.

This analysis was headed by the first author. During the data analysis, the first author had regular meetings with the other two authors to discuss the development of subcategories, categories, and themes. This involved a reflexive process where we sought out positions that challenged our knowledge and prejudice. Bracketing of preconceptions were emphasized in order to draw critical attention to the seclusion cases. The analytic steps are outlined to strengthen trustworthiness [47]. To increase credibility, an external researcher examined similarities within and differences between categories [44].

### Ethical approvals

Each ward selected the case descriptions that were submitted. The patient descriptions were treated confidentially and anonymized by removing any information that could identify the patients. The staff who filled in the forms was also anonymous. The ethical principles of the World Medical Association’s Declaration of Helsinki were followed. The study was approved by the Data Protection Officer at Akershus University Hospital (reg. no. 2012/095). The Regional Committee for Medical and Health Research Ethics (REK) found that the project did not need their approval as it was considered formally as a health services research project based on anonymous data (REK South-East, reg. no. 2013/243).

## Results

The main finding is that *the relationship between treatment and control during seclusion produces ethical challenges*. This theme and the three included categories, each with three subcategories, emerged in the analysis. The participants experienced a sincere desire to provide sound treatment. The descriptions show particularly how taking control of the patient may be difficult and that working under such conditions is burdensome and may put psychosocial strain on staff faced with various ethical challenges. The main results are presented in Table 1. In the text, quotes from participants are presented in italics. Appropriateness and representativeness were emphasized in choosing the quotations [44].

**Table 1:** Overview of main theme, three categories, and nine subcategories

Theme		
The relationship between treatment and control produces ethical challenges when seclusion is used.		
Categories		
The staff has a desire to provide good treatment during seclusion.	The need for control provides treatment dilemmas during seclusion.	It is challenging to work with patients being secluded.
Subcategories		
The staff’s loyalty to the treatment plan is important for performing good seclusion.	Threats and risk of violence make safety a priority over self-determination.	Being ‘on seclusion’ places a psychosocial strain on staff.
A separate seclusion area is important for the quality of treatment during seclusion.	Seclusion is used in conjunction with mechanical restraints.	It is burdensome to work with patients when optimal solutions are lacking.
The staff experiences that patients are mainly negative towards seclusion.	‘Voluntary seclusion’ is coercion without specific legal basis.	Restrictions provide a basis for reflection.

### The staff has a desire to provide good treatment during seclusion

It appears to be an ethical challenge to provide adequate treatment during seclusion. Loyalty to the treatment plan is the code found most often in this category. The subcategories are related to the category because they all describe elements that are important for providing good treatment during seclusion.

### The staff’s loyalty to the treatment plan is important for performing good seclusion

Several of the participants describe the importance of being loyal to what is determined by the treatment group. The staff should act coherently and consistently with the prescribed treatment. A participant describes this attitude with the following words:

‘*This requires us as staff to coordinate performing and decisions that we convey to the patient*’.

Unpredictability would imply confusion and ambivalence both for the patient and the staff and lead to discussions and, at worst, acting out.

*This* [the feeling of lack of predictability] *led to patient insecurity and it seemed like having to deal with different requirements and rules from different staff contributed greatly to acting out and a sense of ambivalence and unrest*.

Many of the participants describe a sensitivity and awareness of how important it is for the patients that the staff agrees on treatment during seclusion.

### A separate seclusion area is important for the quality of treatment during seclusion

Participants describe that the fundamental facilities can be a hindrance to doing a proper job. An important example of a dysfunctional system is a situation when:

‘*the patient is secluded in an ordinary patient room, and seclusion in a separate unit would have been better*’.

When seclusions are carried out in ordinary rooms, with many other patient rooms nearby, the participants state that this often means that staff must ‘*physically hold the door to the room*’. A participant reflects on the extent to which this is suitable for milieu therapy and wonders if seclusion in a room may have no real treatment effect beyond the fact that it prevents the patient from pestering other patients. Another participant states that stimuli restriction is achieved, but with little opportunity for activities. The lack of suitable seclusion conditions is addressed by several participants, and this is identified as an ethical challenge because it is important for quality of treatment. There are also some experiences regarding seclusion in a separate unit, i.e., the patient is taken out of the ordinary environment and moved to a unit where he or she is given a separate room. This allows patients ‘*to move freely in their own room and in the corridor behind a closed door*’, giving them a greater possibility of movement while at the same time maintaining protection and security.

### The staff experiences that patients are mainly negative towards seclusion

The negative experiences are reflected in several descriptions of patients who experienced that seclusion was part of punishment and expressed disagreement.

*‘The patient strongly disagreed to seclusion as treatment*’.

The participants report that some patients expressed their feelings both verbally and through their actions. Others gave only oral expression against seclusion. Other patients acted primarily through physical reactions or behavior.

‘. . *retreated to his own room and was perceived as hostile to staff in the days that followed*’.

The staff reports various observations regarding how patients experience seclusion. They mostly find the patients negative towards seclusion, but they also find that some are positive or neutral. The complexity of some patients’ experiences can be illustrated through this anecdote: A patient expressed that seclusion did not comply with Norwegian law or human rights and ‘*made statements to a great frustration towards a sense of being ruled and controlled as a child*’. However, the patient, at the end of the seclusion, expressed that being secluded made her feel safe and considered it to have positive dimensions.

### The need for control provides treatment dilemmas during seclusion

Various types of control were described by participants. We found the code ‘safety’ most often in this category. The following subcategories are related because they describe different forms of control that affect the ability to provide treatment during seclusion.

### Threats and risk of violence make safety a priority over self-determination

Threats, violence, and patients acting out are part of the daily working life of staff.

‘*He threatened staff but did not physically attack people, only ruined material things’*.

Another writes that ‘*the staff was exposed to patients who attacked them*’. This creates fear; the staff becomes reserved and some feel insecure about those who are acting out. Several participants describe that there is an ethical challenge to weigh threats, violence, and safety against self-determination and good treatment. There are regular reflections and discussions on this theme. Simultaneously, there is an awareness that ‘*the safety aspect must be given priority over the patient’s self-determination*’ because it must be ‘*ensured that the patient does not harm himself or others*’. The risk of violence among patients affects others and safety rules include all patients, e.g., taking shoelaces and belts from all secluded patients. The staff is well aware of this ethical challenge but struggles to find an effective solution.

### Seclusion is used in conjunction with mechanical restraints

A few of the participants mentioned that seclusion is used in conjunction with mechanical restraints (belt bed). For contextual purposes, mechanical belts should only be used towards the patient when this is absolutely necessary to prevent him from harming himself or others, or to avert significant damage to buildings, clothing, fixtures or other items [6]. The most common procedure is to use seclusion after mechanical restraints. The participants reflected on this topic and asked questions: ‘*Is it correct and appropriate to use seclusion after mechanical restraint in bed?’* The alternative they outline is to observe the patient in the environment with other patients. They often conclude that the solution for such a patient would be to seclude him or her in order to observe behavior in calm and controlled conditions. Others seem to be skeptical about whether mechanical restraint was justified.

‘*It this case, it can be discussed whether easier means are well tested. As we have good experience avoiding the use of mechanical restraint with this patient, using female staff, spending enough time and being patient*’.

The participants reflect on when mechanical restraint should be ended: ‘W*ould it be possible to remove the bed restraints while the patients were sleeping?*’ and ‘*discussions about how long the patient is allowed to be fully fixed in bed*’. Some are worried that their relationship with the patient may worsen after an intervention.

‘*Debriefing with the patient afterwards, when available, is therefore an important aspect of restoring/maintaining trust*’.

Most participants describe an ethical awareness of the use of mechanical restraints, although there are some cases where staff does not reflect on this ethical challenge at all.

### ‘Voluntary seclusion’ is coercion without specific legal basis

There is uncertainty and differing opinions regarding whether patients both voluntarily and involuntarily admitted may be secluded over time without any legal decision.

‘*Basically, the culture is that seclusion is a tool that can be used also for patients asking for this’*.

Another writes that it was not necessary to make a formal decision because the patient agreed on the decision. It is mentioned by a participant that the patient has been informed of the possibility of objecting since it is a voluntary seclusion, but the dilemma is that staff is unsure what the patient understands and remembers about the given information.

Another aspect of ‘voluntary seclusion’ is the secluding of patients who are voluntarily admitted. A participant reports precisely about this dilemma:

‘*There was a special challenge in relation to the patient admitted under section 2.1 of the Mental Health Care Act* [Voluntary Section]*, which was simultaneously subject to seclusion*’.

It is mentioned as a specific challenge that, despite voluntary admission, the condition of the patient may be so severe that he/she needs seclusion. The patient is, as a participant succinctly expresses, ‘*voluntarily admitted, but treated as she is involuntary admitted*’.

### It is challenging to work with patients being secluded

Several participants describe that working with secluded patients is challenging in different ways. This topic has emerged in the analysis process as three related subcategories.

### Being ‘on seclusion’ places a psychosocial strain on staff

The participants described that staff became tired, mentally exhausted, and afraid of the close and necessary follow-up required when being with patients in a room or separate unit for seclusion. Some have experienced loneliness in having few people to ask questions. Personnel resources are described as an ethical dilemma. The staff argues that there should be more people at work. How long the staff must work with patients being secluded is discussed. Despite this, ‘*patients could benefit from fewer staff*’, but they are striving to find an optimal solution due to the psychosocial strain on staff. Participants described that it is:

‘*important for the staff with breaks to be able to do a focused and good therapeutic job*’.

The participants agree that there are continuous challenges as the staff’s interests and strains may conflict with the patient’s needs.

### It is burdensome to work with patients when optimal solutions are lacking

Many participants describe that different situations and events during seclusion affect staff. A participant describes that it is stressful to see that the patient is bothered when you do not have an optimal solution.

‘*It was not always easy to stand outside and see how bothered the patient was without being able to find an optimal solution*’.

The staff want to avoid using forced medication or other forms of coercion, and they try to persuade the patient to consent. Despite this, the participants describe situations where staff must physically hold the door to the patient’s room and force medication on patients who resist. The participants describe this as a complex situation in which emotions are involved. They feel that being emotionally touched is positive because it indicates self-reflexivity.

‘*We think it is positive to be touched. It shows that we have a good stance and that we are reflecting on what we do with our patients*’.

This involves being aware of the power relationship between the patient and staff in mental health care.

‘*We are aware that we have some power in our job and it is absolutely necessary that we do not abuse it*’.

The staff describes seclusion as an ethical challenge that affects them emotionally, while at the same time they describe having a basically good ethical attitude from which to address this challenge.

### Restrictions provide a basis for reflection

There are several types of limitations described. First, seclusion itself is a limitation because it often involves stimulus reduction. This leads to:

‘*ongoing and continuous discussion about stimulus-limiting measures, as it can easily become too few activities when the patient is secluded*’.

The participants reflect on whether increased unrest really is associated with increased stimuli. A more specific case is described: ‘*Enjoyable activity such as table tennis was not allowed*’. The staff finds it difficult to argue for these types of limitations. Furthermore, participants describe restrictions on access to phones as well as on the opportunity to go for a walk or receive visits. There may be partial limitations or no access whatsoever. Some of the participants reflect on how drastic it is to restrict a patient’s freedom of movement, while others consider that the patient profits by limiting stimuli from the outside world.

## Discussion

### The need for control creates treatment dilemmas under seclusion

This study found that balancing between treatment and control implied several ethical challenges for the staff. One of these was that controlling violent behavior is part of the daily working life of staff. Violent and aggressive events occur commonly in mental health settings; this is supported by earlier research findings [48, 49]. A Norwegian study shows that 31% of all inpatient and 59% of involuntarily committed inpatients (34% of all inpatients) had a risk of violent behavior [50]. We found that violent behavior made it necessary for staff to weigh threats, violence, and safety against self-determination, and they experienced this to be an ethical challenge. This experience is supported by earlier research findings about management of patients’ aggressive behavior and ethical dilemmas, i.e., they find it challenging to balance the patients’ best interests and those of other people when making decisions on whether or not to seclude [18].

The weighing of threats, violence, and safety may lead to the use of mechanical restraints. The Norwegian Parliamentary Ombudsman has reported that coercive measures are used often during seclusion [51]. This supports our finding that seclusion is used in conjunction with mechanical restraints. However, to our knowledge, there is no study supporting that seclusion is used frequently after mechanical restraint. The participants in our study argue for the use of seclusion after restraint in order to observe behavior in calm and controlled conditions. Being with the patient in a calm environment after use of mechanical restraints seems reasonable, taking into account that subjective distress may occur after restraint use and that contact with staff is most helpful in alleviating that distress [52].

The weighing of threats, violence, and safety can be even more ethically challenging when voluntarily admitted patients are secluded and when patients are secluded without an administrative decision being made. Seclusion among voluntarily admitted patients seems to vary [31, 53], and legal status is not a sure indication of the experience of coercion [53–55]. Patients who are not formally subjected to coercion may still experience perceived coercion. This is in line with our finding of *voluntary seclusion as coercion without legal basis*. It is a fundamental principle of the law that patients who admit themselves voluntarily to a hospital should be able to leave when they wish, as far as they do not constitute an obvious and serious risk to their own life and health or those of others [6]. This also applies if the patient is being secluded. However, being secluded is a form of coercion, and patients may be afraid of being subject to further coercion. Hence, they may choose to obey staff despite their wish to leave the hospital. Secluding the patient without an administrative decision makes the situation even more challenging. This is a violation as the patient loses legal rights such as the right to an appeal and judicial review of the decision.

Threats and risk of violence seem to have a serious impact on staff; they become frightened, reserved, and insecure. These reactions are important to take into consideration as these feelings may affect the quality of treatment. The staff members in our study do regular reflections and debriefing with the patients after mechanical restraint. A recent pilot study suggests that a post-seclusion review may reduce the use of seclusion, as well as time spent in seclusion, and have positive effects on the therapeutic relationship [56]. Post-seclusion interventions should focus on changing the seclusion practice through reflexive approaches including both patients and staff [57]. In Norway, patients are now offered the opportunity to have a dialogue about their experience after an episode of coercion [6]. This systematic routine, we believe, will improve the quality of the services.

### It is challenging to work with patients being secluded

Our findings illustrate that working with patients being secluded is stressful due to lack of adequate solutions, and staff become tired, scared, and mentally exhausted from the psychosocial strain. This is comparable to earlier research that found distressing emotions of uneasiness, fear, arousal, numbness, anxiety, and guilt in staff’s descriptions of restraint and seclusion [18, 36, 58].

As seclusion may result in deleterious physical and psychological effects on staff [59], a reasonable interpretation is that these may also affect the treatment environment. These factors are proven to affect both treatment outcomes and patient satisfaction [60, 61]. All these dimensions may also be considered ethical, and it should be discussed to what extent such a treatment environment is professionally justifiable. The staff seems to reflect on these challenges, but they are striving to find an optimal solution that takes into account both staff and the patients’ views. They argue for more people at work and emphasize the importance of being able to take breaks. Increasing staff-to-patient ratios is mentioned in a review as one of several interventions for reducing the use of seclusion as it can allow staff to provide more sensitive care and a safer environment for both staff and patients [22]. Longer work experience and more variability in work experience among staff is also associated with less seclusion [62].

It was also challenging to work with patients being secluded because the patients mainly responded negatively to the practice. This is supported in several previous studies where patients perceived seclusion to be a distinctly negative experience [32–35]. Some recent studies suggest that patients can experience seclusion ambiguously—both beneficial and not—as having access to shelter or as being sent to prison [63, 64].

### The challenge of providing good treatment during seclusion according to ethical principles

It might be helpful to formulate the different ethical dilemmas experienced by the professionals as conflicts between ethical principles. The four traditional principles of medical ethics provides a framework and a language in which to articulate the values at stake and the value conflicts that arise [65].

This study shows that it is ethically challenging to promote a therapeutic approach when it must be balanced against the need for control in the form of seclusion, e.g., when violent behaviour makes it necessary for the staff to balance threats, violence, and safety against self-determination. A decision here should involve considerations of the violent patients’ right to autonomy versus considerations of the risk of inflicting harm towards other patients’ or staff, including the risk of harm the seclusion may imply for the violent patient. This is consistent with a recent review finding that patient autonomy is a fundamental challenge facing any use of coercion and that a primary ethical challenge when coercion is used is to assess the balance between promoting good (beneficence) and inflicting harm (non-maleficence) [40]. The fundamental challenge of patient autonomy is supported in a fair amount of earlier research emphasizing that people with mental health problems want to be actively involved in decisions about their care [66–71]. When the staff and the patient disagree on the treatment, it is important to not only consider the patient as irrational, but explicitly discuss the ethical principles.

In this study, the principle of beneficence may conflict with autonomy when staff wants to use seclusion as treatment when the patient does not desire it. Beneficence includes a moral obligation to act for the benefit of the patient. However, patient preferences should be acknowledged, and staff should weigh all available options and carry out seclusion only when the benefits exceed the disadvantages [72]. One should then know that seclusion as treatment is effective enough to outweigh the disadvantages of acting against the patient’s desire. Unfortunately, there are, to our knowledge, no studies that definitively support the therapeutic effect of seclusion [1, 2, 36–38, 73]. Hence, it is difficult to find sufficient ethical arguments for the implementation of seclusion against the patient’s will. Following this line of reasoning, one could argue that the principle of autonomy should be prioritized. However, it may also be argued that the disadvantage for the other patients is so great in not using seclusion that ‘do no harm’ (non-maleficence) to the other patients should be prioritized, i.e., a patient needing to be secluded could be so disruptive and chaotic that he/she impedes the recovery process for other patients. A treatment situation like this might also conflict with the principle of justice as fairness, although justice is for every individual, not just the patient and will therefore have to take into consideration broader perspectives [65].

The principle of autonomy is challenged when restrictions are imposed against the patient’s will, e.g., regarding visits, the opportunity for walking, fresh air, telephone usage, and other activities. This seems to be based on the ideology (of controversial research basis [1, 2, 36–38]) that stimulus reduction is useful for patients being secluded. However, several of the staff members are doubtful about the therapeutic effect of restrictions; they want to act for the patients’ benefit and have a focus on avoiding harm, but they are trapped in a system that limits patients’ autonomy. Do no harm” is obviously a good principle; but breaches of the principle may be justified, as it could be legitimately argued that the long-term benefits would outweigh the short. Other examples are found when participants describe that seclusion is often conducted in rooms, while staff argues for greater freedom of movement using a seclusion unit. A separately locked PICU is an alternative to seclusion and has been proven to have favorable effects on behaviors associated with and the actual numbers of violent and threatening incidents [73]. The possibility of facilitating autonomy may be considered greater in a seclusion-suitable unit than by seclusion in an ordinary room. Regardless of the structural conditions, the principle of justice confers a moral obligation on staff to implement seclusion in a manner that ensures the highest degree of autonomy for the patient.

We found that staff performs ethical assessments on a daily basis, paying attention to the patient, colleagues, and the health care system. The legitimate use of control is a fundamental responsibility and a key to ethical practice and professional integrity [74]. A recent systematic review of interventions for reducing seclusion found that systematic evaluation of aggressive behavior in patients admitted to an acute psychiatric ward and counselling for staff in high security wards may reduce the use of seclusion [75]. The review concluded that more research is needed to draw more robust conclusions. Another systematic review found that seclusion reduction programs enhance the quality and safety of care [76]. Applying sensory modulation to mental health inpatient care may also reduce the use of seclusion [77, 78]. We will also emphasize the importance of systematizing ethical reflection to face the ethical challenges that staff encounters every day. Moral case deliberation may improve the overall ethical quality of the organization, e.g., by facilitating reflection on good care in general and seclusion in particular [79].

### Strengths and limitations

Concerning the method of this study, the data are based on what clinical staff members write when they answer open questions about ethical aspects of seclusion. This contrasts with a questionnaire consisting of closed, predefined questions. In this way, we allow the health professionals to set the agenda. The positive outcome of this is that we can obtain information about what matters to staff, although at the same time it can be easier to hide experiences they would have been ‘forced’ to consider during an interview.

Data from semi-structured forms are not as robust as interview data. They can easily become superficial and ambiguous and should therefore be interpreted with considerable reservations [80]. Using content analysis effectively on open-ended questions requires that answers are not too brief [43]. The length of the descriptions varied, but most were rich with information of good quality, and time was used to familiarize ourselves with the material and to make a thorough analysis [47]. Representative quotes from transcribed texts are presented to show a connection between the data and the results [44].

A potential bias in this study is that the researchers foreknowledge may have affected the ability to research the topic thoroughly. I.e. preconceptions and assumptions about the research topic may unconsciously have been brought into the research process and made the researchers less open to the participants’ understanding and meaning [81]. We tried to reduce this bias by bracketing, i.e. our preconceptions were temporarily suspended in an attempt to not influence the participants description of the phenomena. This is challenging as it can be impossible to suspend preconceptions totally, especially those one are unaware of [82]. We believe that a constant reflective process challenging foreknowledge and working to bring preconceptions to the level on consciousness was of help.

The fact that data collection was carried out by clinical staff with various experience increases the possibility of shedding light on the research questions from a variety of perspectives [83]. Our assessment is that theme and categories cover the material well and to our knowledge, no relevant data have been systematically excluded or irrelevant data included. This strengthens the credibility [44]. Involving an independent researcher was not to verify, but to determine if an external researcher would agree with the way our data were labelled and sorted [44]. The independent researcher had valuable contributions to the names of the categories, specific input that made us see the material from a new perspective and were essentially in agreement with us regarding how the categories were labelled and sorted. A large amount of material was analysed, giving a deep and rich understanding of health professionals’ ethical challenges during seclusion. The findings may be transferable to psychiatric hospitals with similar seclusion contexts.

## Conclusion

This study is to our knowledge the first health service study on staff’s experiences of the ethical dimensions of seclusion; their main ethical challenge is the relationship between treatment and control when seclusion is used. The staff has a desire to provide good treatment during seclusion. The need for control produces treatment dilemmas, and working with patients during seclusion induces psychosocial strain.

The clinical implications are that the findings may contribute to a better understanding of how to use seclusion in a better way as well as when not to use it.

There is a need for more research to assess the various types of seclusion in the health services, including design of places to implement seclusion, research on less intrusive alternatives to seclusion, and what consequences the psychosocial stress of working with seclusion have for staff.

## Data Availability

The qualitative data or parts of the data may be considered available from the project manager Torleif Ruud on reasonable request. Contact details: torleif.ruud@medisin.uio.no

## Abbreviations

OPCAT: The UN optional protocol to the Convention against Torture
PICU: Psychiatric intensive care unit
